# Supplementary material for: A monocyte-centered framework for predicting immunochemotherapy efficacy in lung squamous cell carcinoma patients
Source: EMBO Mol Med. 2026 Mar 30;18(5):1812–35. doi: 10.1038/s44321-026-00410-y (PMC13179367; doi:10.1038/s44321-026-00410-y)
Supplement: Supplementary file 1 — Appendix [file 44321_2026_410_MOESM1_ESM.pdf]

Table of contents:

Appendix Fig S1	2
Appendix Fig S2	3
Appendix Fig S3	4

Appendix Figure S1. Boxplot and statistical comparison of cell-type proportion

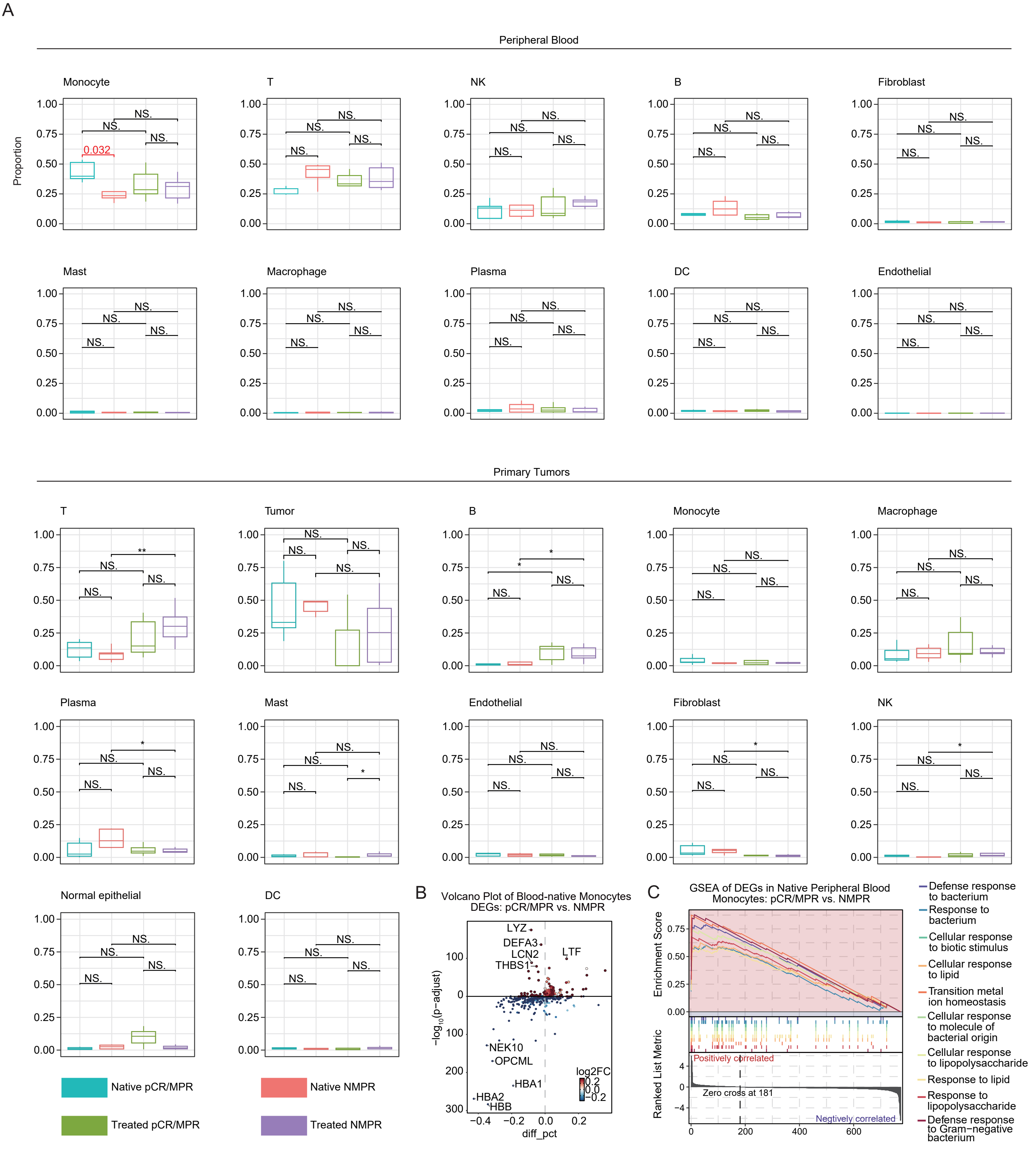

(**A**) Box plot and statistical comparison of cell-type proportion among different response groups using two-sided Wilcoxon test (blood sample n_{blood}=23, before treatment $n_{pCR/MPR}$=5, n_{NMPR}=4, after treatment $n_{pCR/MPR}$=7, n_{NMPR}=7; tumor sample n_{tumor}=26, before treatment $n_{pCR/MPR}$=7, n_{NMPR}=5, after treatment $n_{pCR/MPR}$=7, n_{NMPR}=7; NS: no significant difference, *p < 0.05, **p < 0.01, ***p < 0.001). Box plots followed the Tukey style (Centre line: median. Box bounds: 25th and 75th percentiles. Whiskers: extending to the most extreme data points within 1.5 times the interquartile range (IQR) from the box bounds. Outliers: points beyond the end of the whiskers). (**B**) Volcano plot of differentially expressed genes (DEGs) with pCR/MPR versus NMPR monocytes in native blood; the x-axis indicates percentage differences in gene-expressing cells between two compared groups, and the y-axis shows -$\log_{10}$(adjusted p-value). Dots are colored based on $\log_2$(fold-change) ($\log_2$FC) values. Two-sided Wilcoxon test was utilized to calculated p-values and p-values were adjusted by Bonferroni correction. (**C**) GSEA plot of top 10 upregulated pathways based on DEGs in Appendix Fig.S1B.

Appendix Figure S2. Subpopulation proportion comparison of monocytes, B cells, fibroblasts and macrophages

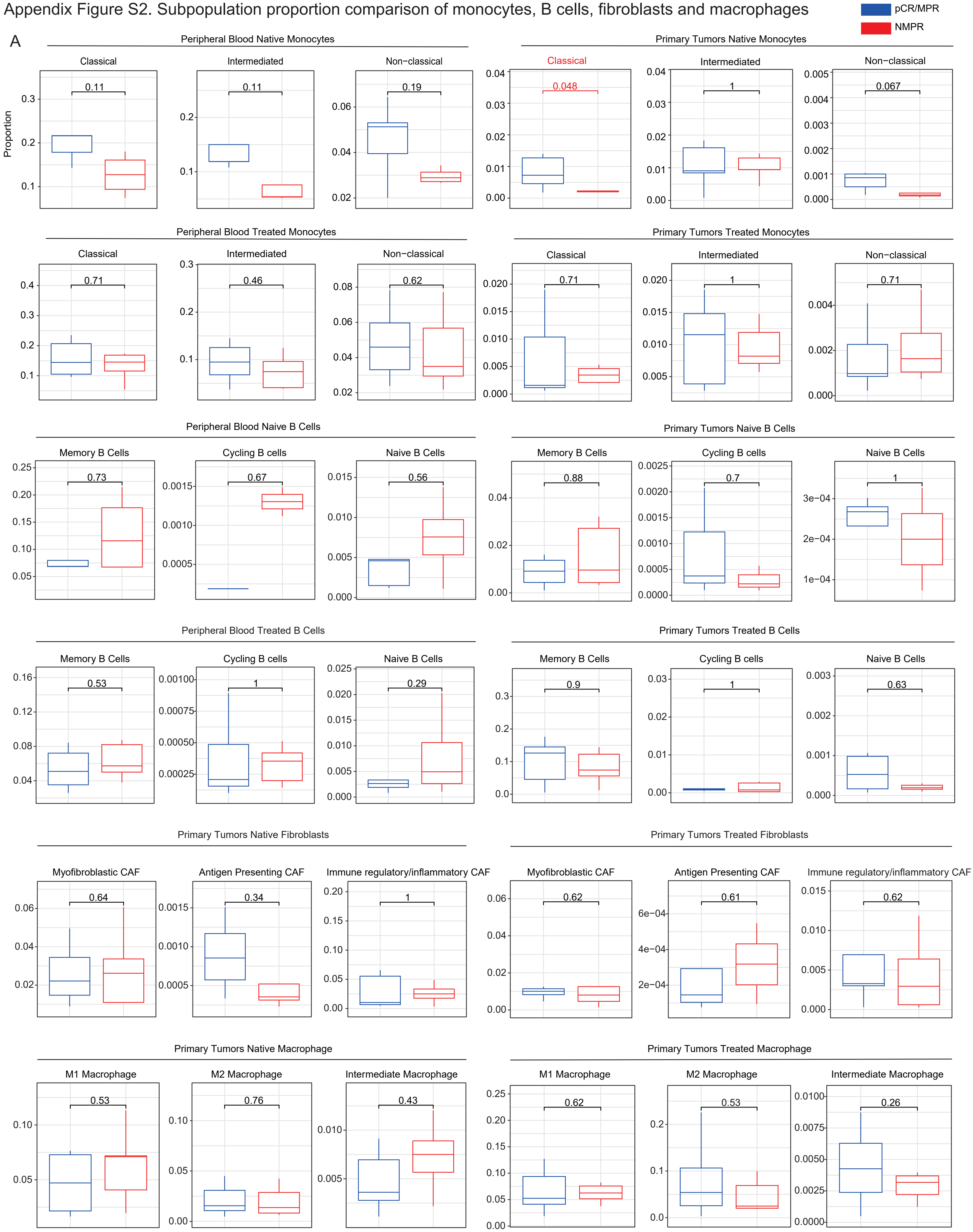

(**A**) Subpopulation proportion box plot and comparison of monocytes, B cells, fibroblasts and macrophages among different response groups (blood sample n_{blood}=23, before treatment $n_{pCR/MPR}$=5, n_{NMPR}=4, after treatment $n_{pCR/MPR}$=7, n_{NMPR}=7; tumor sample n_{tumor}=26, before treatment $n_{pCR/MPR}$=7, n_{NMPR}=5, after treatment $n_{pCR/MPR}$=7, n_{NMPR}=7; CAF: Cancer-associated fibroblast; two-sided Wilcoxon test, significant p-value < 0.05). Box plots followed the Tukey style (Centre line: median. Box bounds: 25th and 75th percentiles. Whiskers: extending to the most extreme data points within 1.5 times the interquartile range (IQR) from the box bounds. Outliers: points beyond the end of the whiskers).

Appendix Figure S3. Subpopulation proportion comparison of plasma and T cells

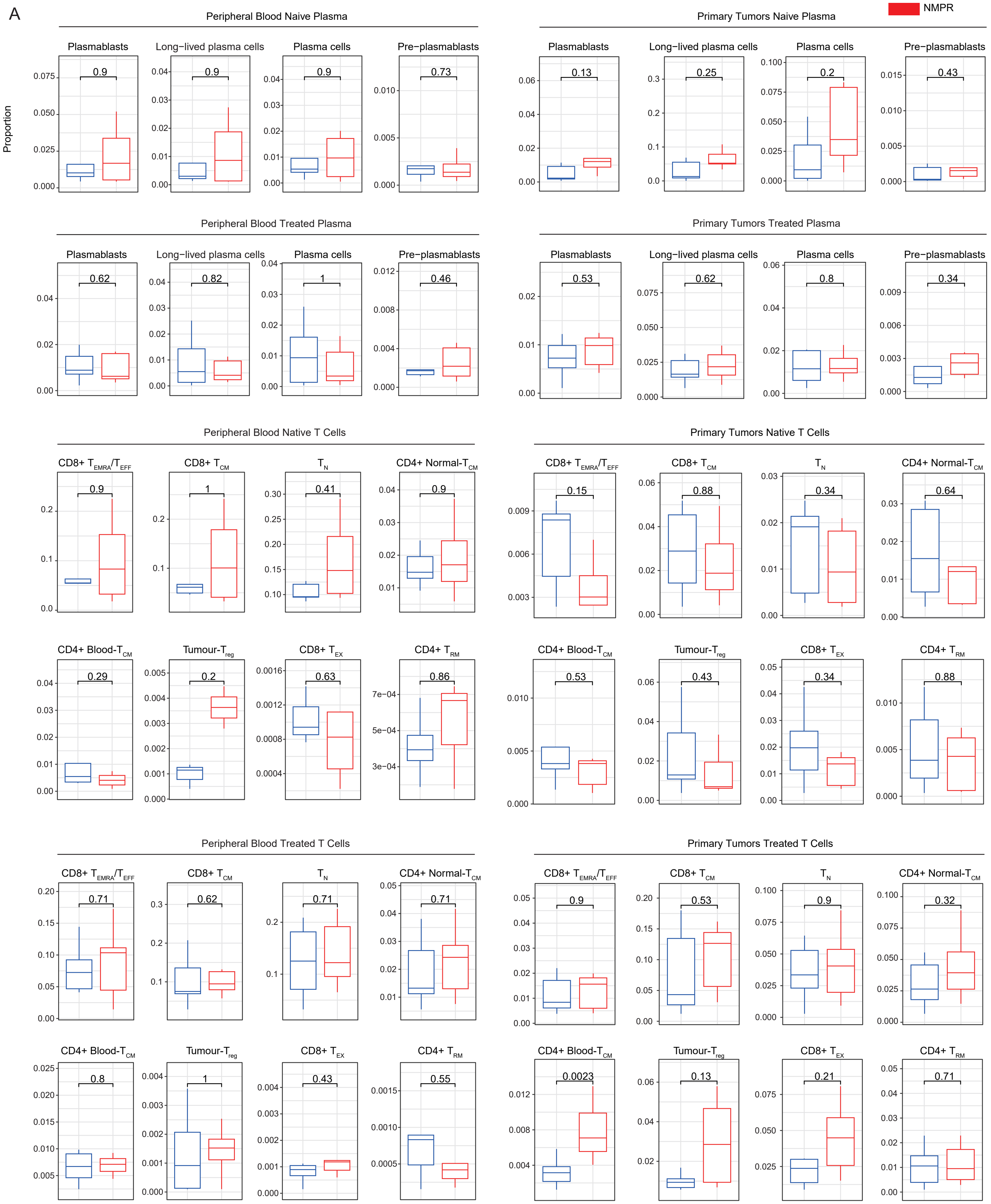

(**A**) Subpopulation proportion box plot and comparison of plasma and T cells among different response groups (blood sample n_{blood}=23, before treatment $n_{pCR/MPR}$=5, n_{NMPR}=4, after treatment $n_{pCR/MPR}$=7, n_{NMPR}=7; tumor sample n_{tumor}=26, before treatment $n_{pCR/MPR}$=7, n_{NMPR}=5, after treatment $n_{pCR/MPR}$=7, n_{NMPR}=7; CD8+ T_{EMRA}/T_{EFF}: effector memory or effector T cells; CD8+ T_{CM}: CD8+ central memory T cells; T_N: naive T cells; CD4+ Normal-T_{CM}: CD4+ Normal-central memory T cells; CD4+ Blood-T_{CM}: CD4+ Blood-central memory T cells; Tumor-T_{reg}: tumor-infiltrating T regulatory cells; CD8+ T_{EX}: exhausted CD8+ T cells; CD4+ T_{RM}: tissue-resident memory T cells; two-sided Wilcoxon test, significant p-value < 0.05). Box plots followed the Tukey style (Centre line: median. Box bounds: 25th and 75th percentiles. Whiskers: extending to the most extreme data points within 1.5 times the interquartile range (IQR) from the box bounds. Outliers: points beyond the end of the whiskers).
