# Supplementary material for: A monocyte-centered framework for predicting immunochemotherapy efficacy in lung squamous cell carcinoma patients
Source: EMBO Mol Med. 2026 Mar 30;18(5):1812–35. doi: 10.1038/s44321-026-00410-y (PMC13179367; doi:10.1038/s44321-026-00410-y)
Supplement: Supplementary file 3 — Table EV2 [file 44321_2026_410_MOESM3_ESM.pdf]

| Characteristic | Total Patients (N=15) | Responders (n=8) | Non-responders (n=7) |
|---|---|---|---|
| **Age, years** | | | |
| Median (Range) | 63 (53-72) | 62 (53-72) | 67 (56-72) |
| **Gender, n (%)** | | | |
| Male | 15 (100.0) | 8 (100.0) | 7 (100.0) |
| Female | 0 | 0 | 0 |
| **Pathological Type, n (%)** | | | |
| Lung Squamous Cell Carcinoma (LUSC) | 15 (100.0) | 8 (100.0) | 7 (100.0) |
| **Smoking Status, n (%)** | | | |
| Smoker | 14 (93.3) | 7 (87.5) | 7 (100.0) |
| Non-smoker | 1 (6.7) | 1(12.5) | 0 |
| **Tumor Stage, n (%)** | | | |
| IIB | 3 (20.0) | 1(12.5) | 2 (28.6) |
| IIIA | 5 (33.3) | 4 (50.0) | 1 (14.3) |
| IIIB | 7 (46.7) | 3 (37.5) | 4 (57.1) |
| **PD-L1 Expression, n (%)** | | | |
| <1% | 1 (6.7) | 0 | 1 (14.3) |
| 1-50% | 3 (20.0) | 1 (12.5) | 2 (28.6) |
| >50% | 2 (13.3) | 2 (25.0) | 0 |
| Unknown | 9 (60.0) | 5 (62.5) | 4 (57.1) |
| **Treatment Cycles, n (%)** | | | |
| 2 cycles | 12 (80.0) | 7 (87.5) | 5 (71.4) |
| 3 cycles | 3 (20.0) | 1 (12.5) | 2 (28.6) |
| **Treatment Regimen, n (%)** | | | |
| Nab-paclitaxel+Carboplatin+Pembrolizumab | 1 (6.7) | 1 (12.5) | 0 |
| Nab-paclitaxel+Carboplatin+Sintilimab | 7 (46.7) | 4 (50.0) | 3 (42.9) |
| Nab-paclitaxel+Carboplatin+Toripalimab | 6 (40.0) | 3 (37.5) | 3 (42.9) |
| Nab-paclitaxel+Cisplatin+Sintilimab | 1(6.7) | 0 | 1(14.3) |

Table EV2. The Clinical Characteristic Distribution Table of scRNA-seq Cohort. This table is a summary of clinical information of patients who contribute samples for scRNA-seq. The data summarized based on Table EV1.

Age: The age of patients.

Gender: The gender of patients.

Pathological Type: The cancer type of patients.

Smoking Status: The patients with smoking index higher than 0 were labeled as smoker and others were labeled as non-smoker.

Tumor Stage: The stage information of tumor.

PD-L1 Expression: The expression rate of PD-L1 in tumors.

Treatment Cycles: The treatment cycles of patients.

Treatment Regimen: The treatment regimen used for patients.
