# Supplementary material for: A monocyte-centered framework for predicting immunochemotherapy efficacy in lung squamous cell carcinoma patients
Source: EMBO Mol Med. 2026 Mar 30;18(5):1812–35. doi: 10.1038/s44321-026-00410-y (PMC13179367; doi:10.1038/s44321-026-00410-y)
Supplement: Supplementary file 7 — Table EV6 [file 44321_2026_410_MOESM7_ESM.pdf]

Characteristic	Patients of Complete Blood Count (N=228)	Patients of Tumor Slides (N=54)
**Sampling Year, n (%)**		
2022	11 (4.8)	0(0)
2023	111(48.7)	12(22.2)
2024	106 (46.5)	42 (77.8)
**Age, years**		
Median (Range)	66 (43-82)	67(53-78)
**Gender, n (%)**		
Male	224(98.2)	54(100.0)
Female	4(1.8)	0(0)
**Pathological Type, n (%)**		
Lung Squamous Cell Carcinoma (LUSC)	228 (100.0)	54 (100.0)
**Response Status, n (%)**		
Pathologic Complete or Major Responses	181(79.4)	33(61.1)
Non-Major Pathological Response	47(20.6)	21(38.9)

Table EV6. The Clinical Characteristic Distribution Table of Validation Cohort. This table is a summary of clinical information of patients who contribute samples for CBC and IF samples. The data summarized based on Table EV4 and EV5.
Sampling Year: The collection year of samples.
Age: The age of patients.
Gender: The gender of patients.
Pathological Type: The cancer type of patients.
Response Status: The response of patients after treatment.
