# Supplementary figures and images for: A monocyte-centered framework for predicting immunochemotherapy efficacy in lung squamous cell carcinoma patients
Source: EMBO Mol Med. 2026 Mar 30;18(5):1812–35. doi: 10.1038/s44321-026-00410-y (PMC13179367; doi:10.1038/s44321-026-00410-y)

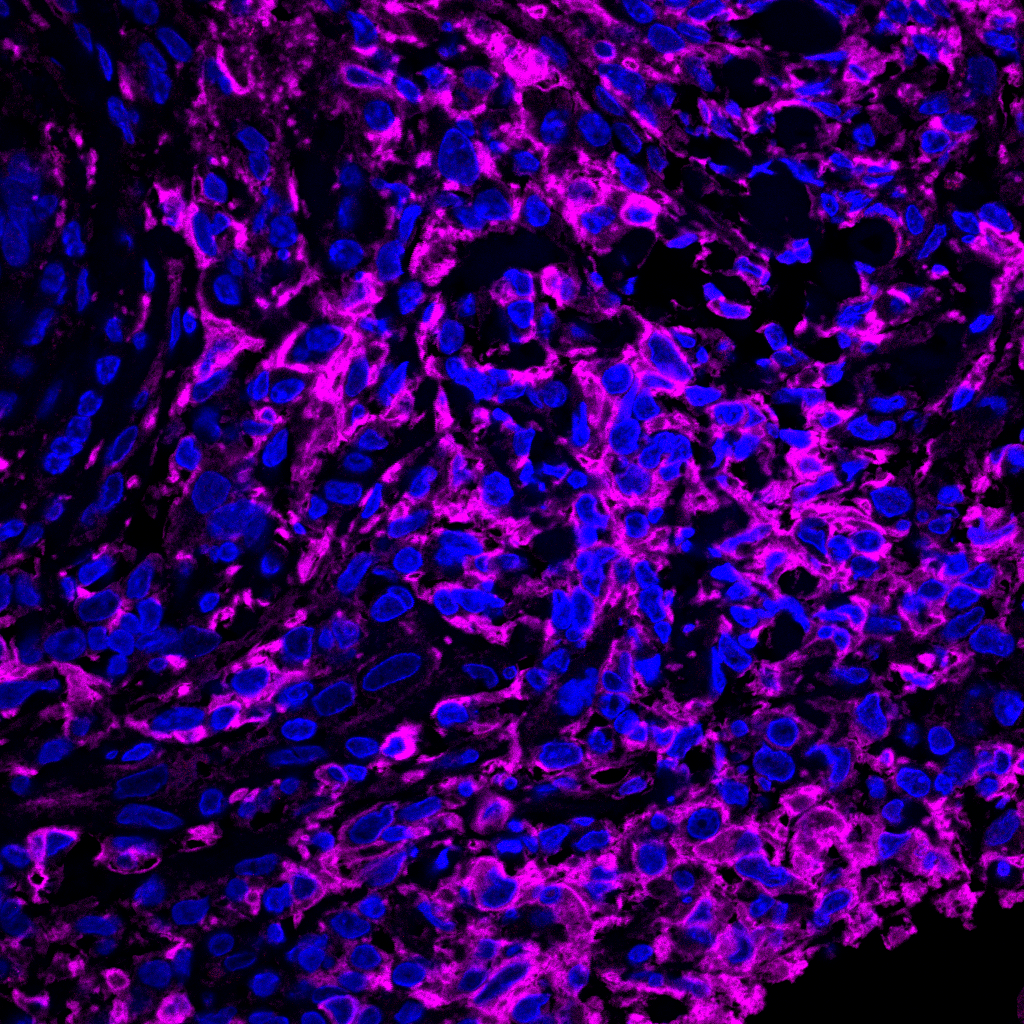

Supplement: Supplementary file 13 — Source data Fig. 2 [file 44321_2026_410_MOESM13_ESM.zip › Figure 2/2E/Representative Figure/pCR/pCR_c1+2+4.tif]

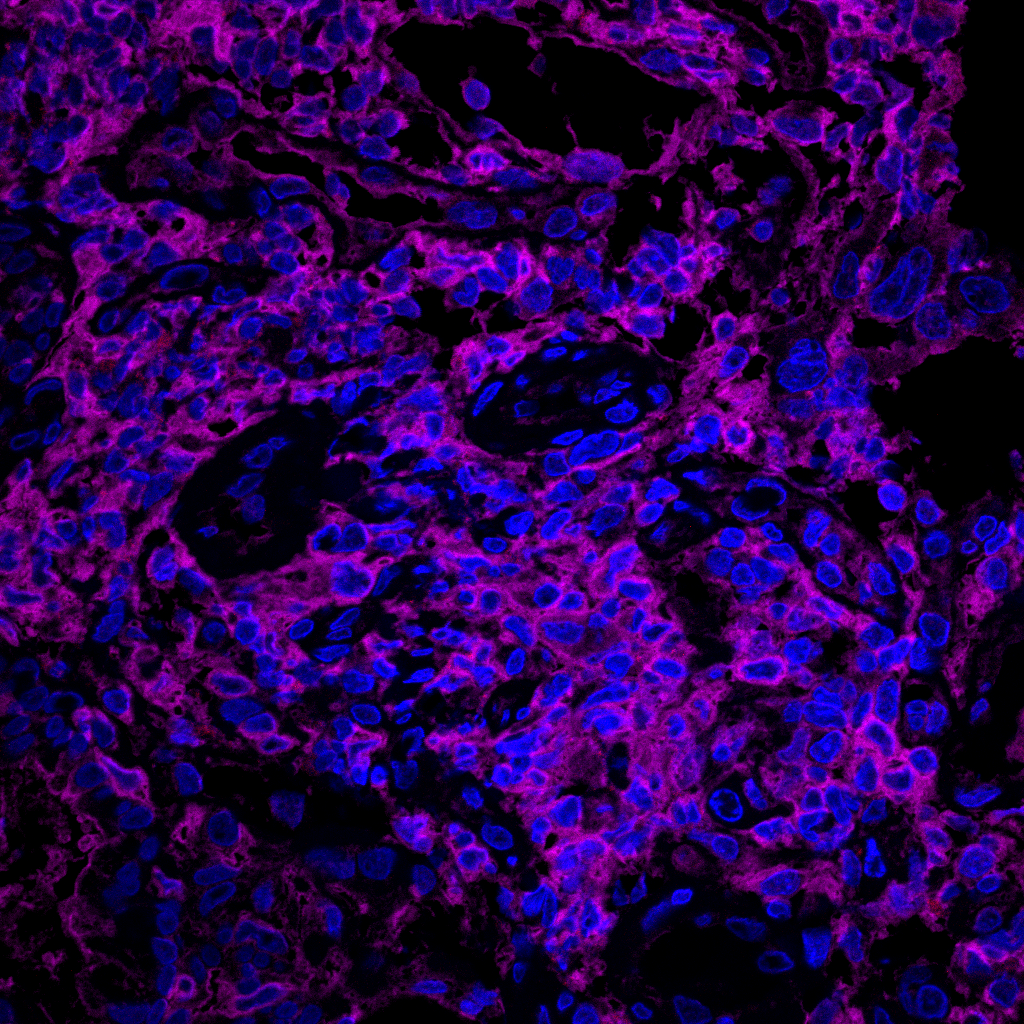

Supplement: Supplementary file 13 — Source data Fig. 2 [file 44321_2026_410_MOESM13_ESM.zip › Figure 2/2E/Representative Figure/MPR/MPR_c1+2+4.tif]

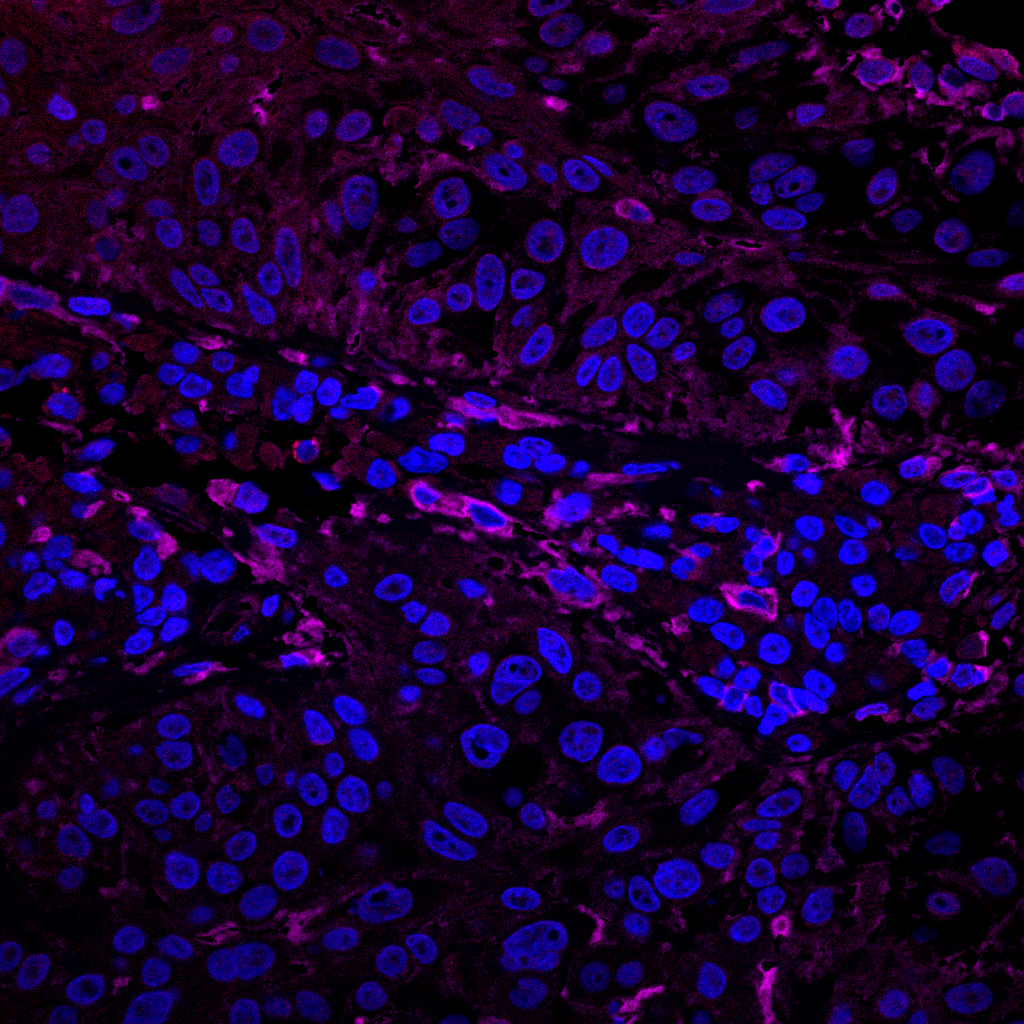

Supplement: Supplementary file 13 — Source data Fig. 2 [file 44321_2026_410_MOESM13_ESM.zip › Figure 2/2E/Representative Figure/NMPR/NMPR_c1+2+4.tif]

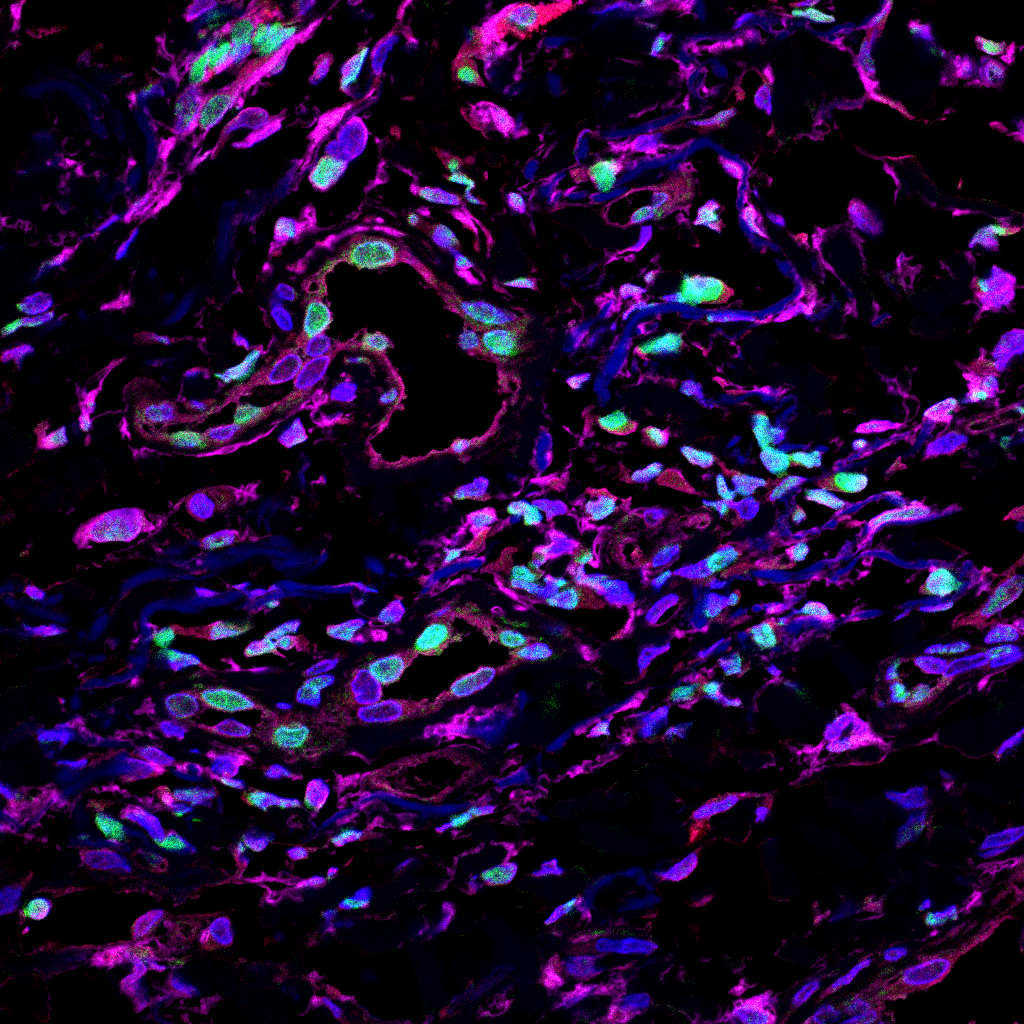

Supplement: Supplementary file 14 — Source data Fig. 3 [file 44321_2026_410_MOESM14_ESM.zip › Figure 3/3E/Representative Figure/15%/15%_c1-4.tif]

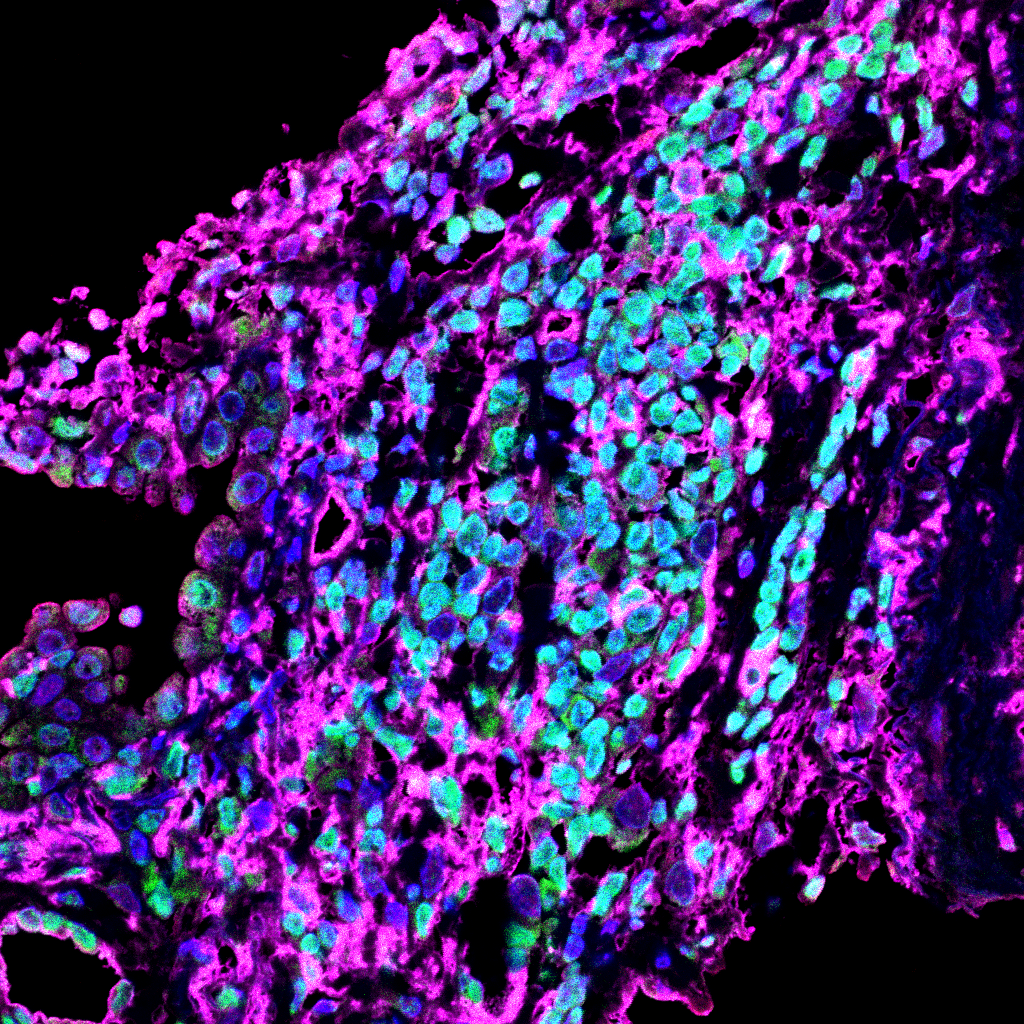

Supplement: Supplementary file 14 — Source data Fig. 3 [file 44321_2026_410_MOESM14_ESM.zip › Figure 3/3E/Representative Figure/5%/5%_c1-4.tif]

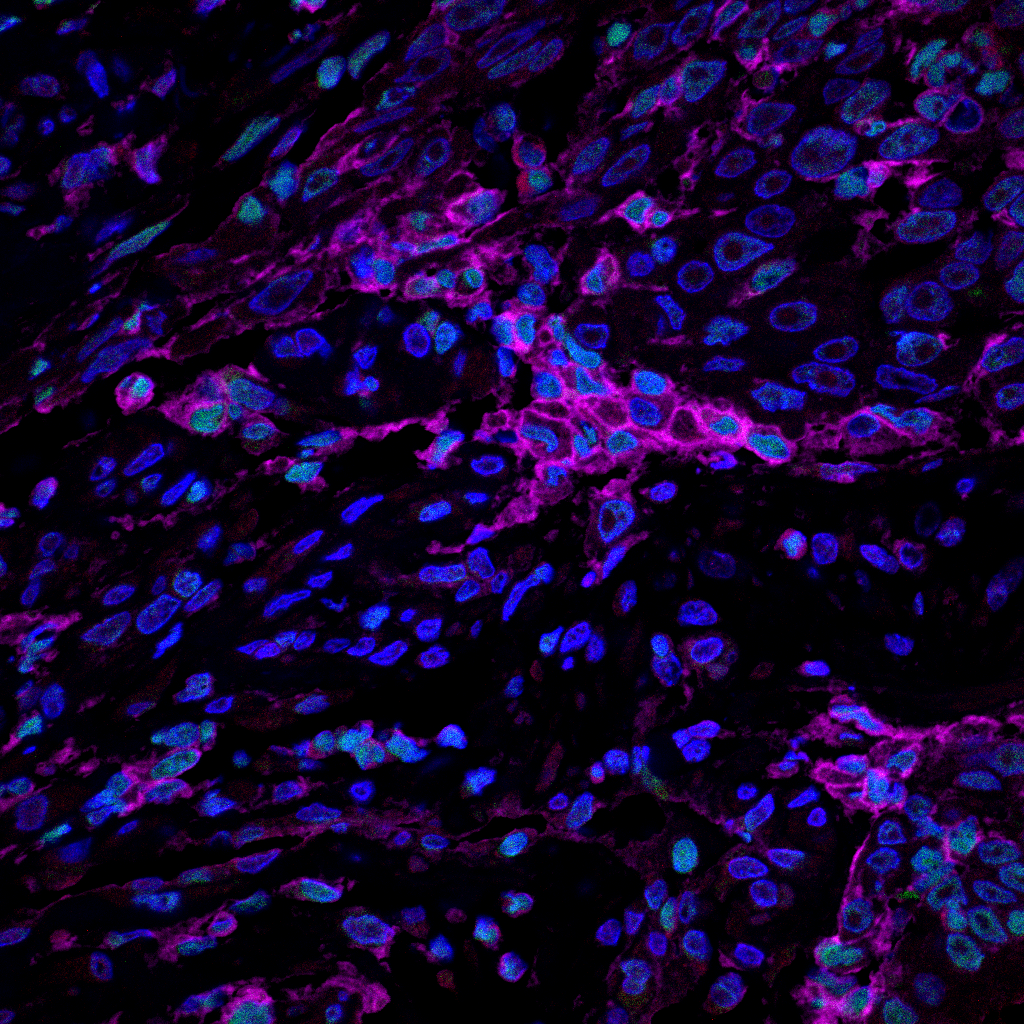

Supplement: Supplementary file 14 — Source data Fig. 3 [file 44321_2026_410_MOESM14_ESM.zip › Figure 3/3E/Representative Figure/30%/30%_c1-4.tif]

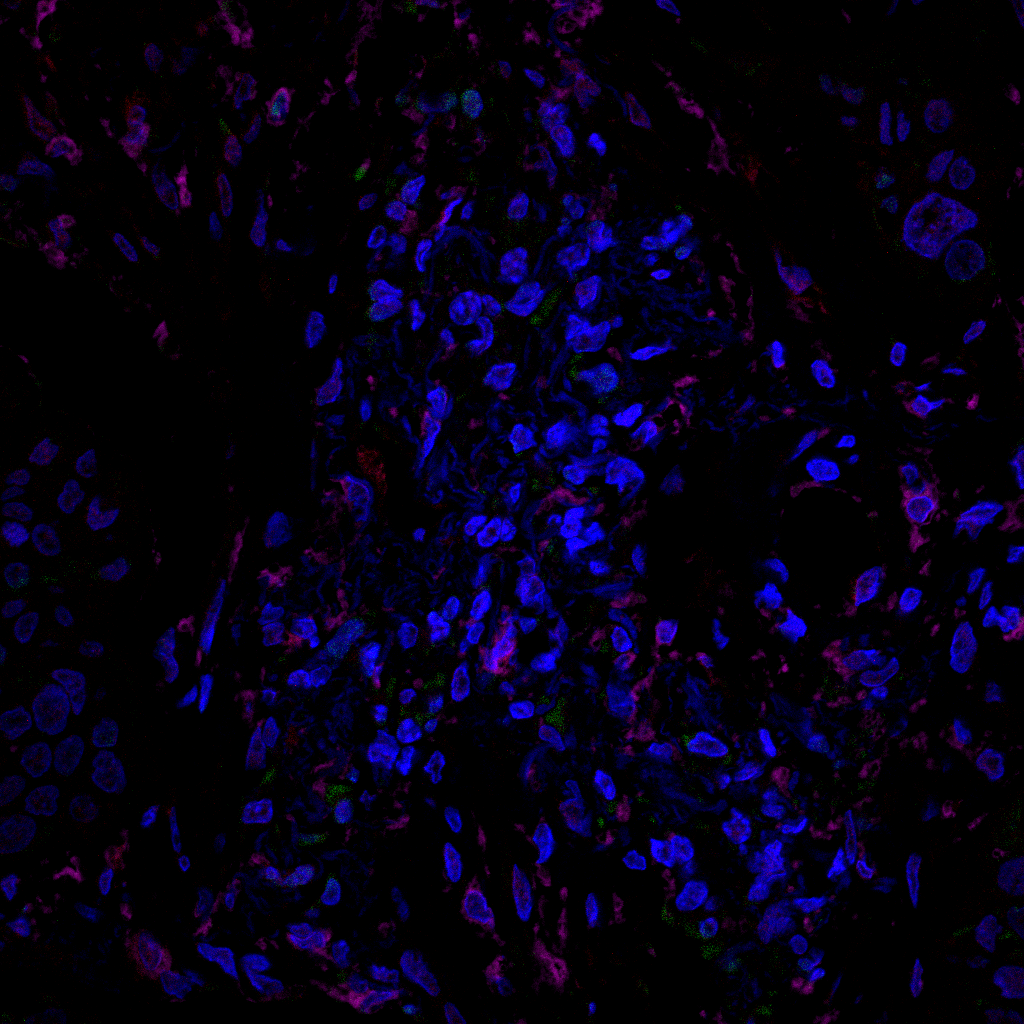

Supplement: Supplementary file 14 — Source data Fig. 3 [file 44321_2026_410_MOESM14_ESM.zip › Figure 3/3E/Representative Figure/95%/95%_c1-4.tif]

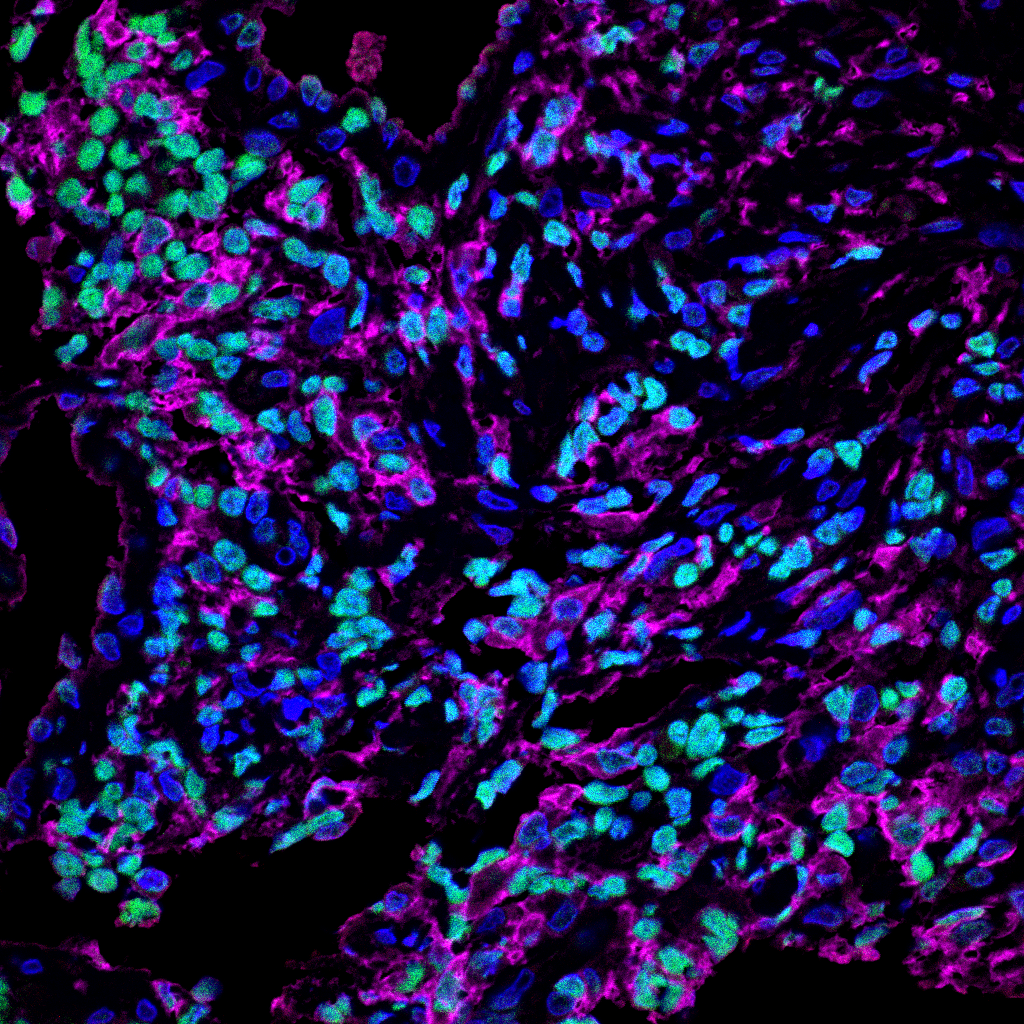

Supplement: Supplementary file 14 — Source data Fig. 3 [file 44321_2026_410_MOESM14_ESM.zip › Figure 3/3E/Representative Figure/0%/0%_c1-4.tif]

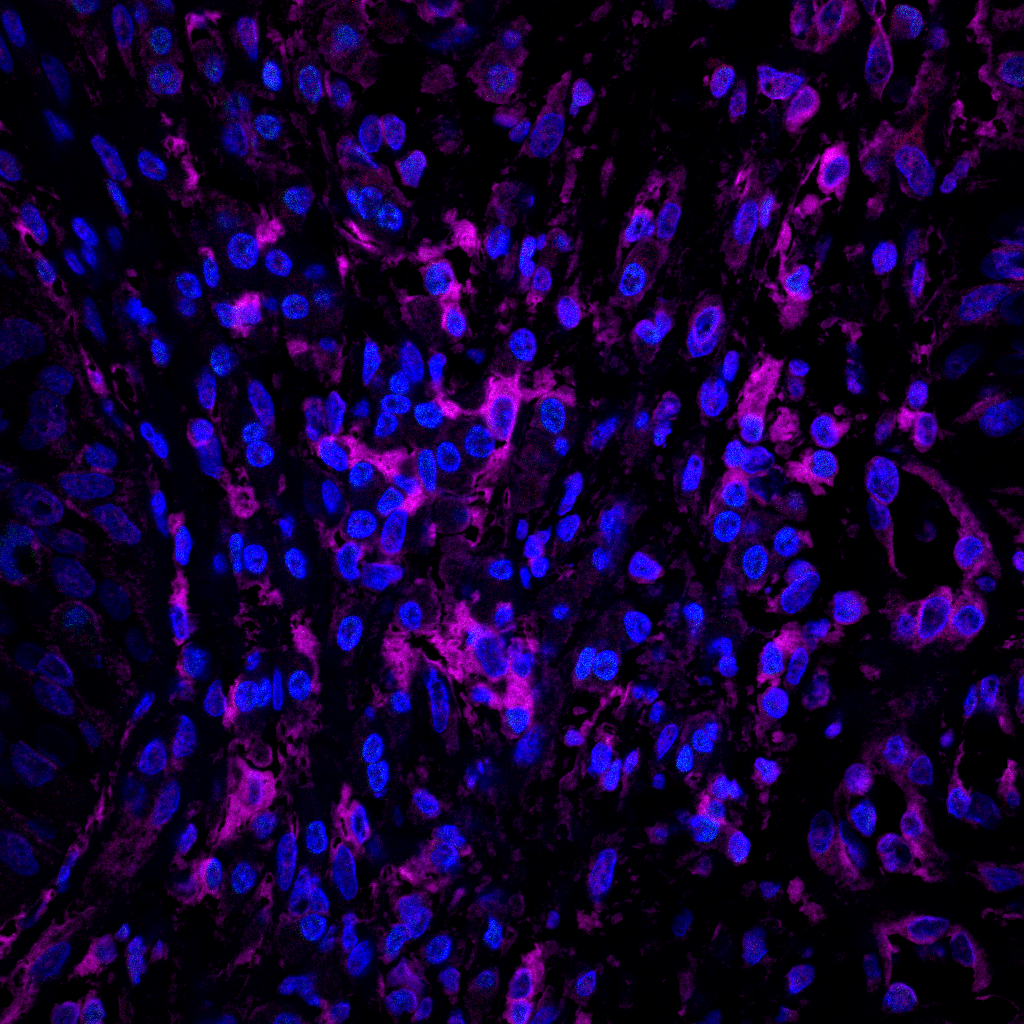

Supplement: Supplementary file 14 — Source data Fig. 3 [file 44321_2026_410_MOESM14_ESM.zip › Figure 3/3E/Representative Figure/50%/50%_c1-4.tif]

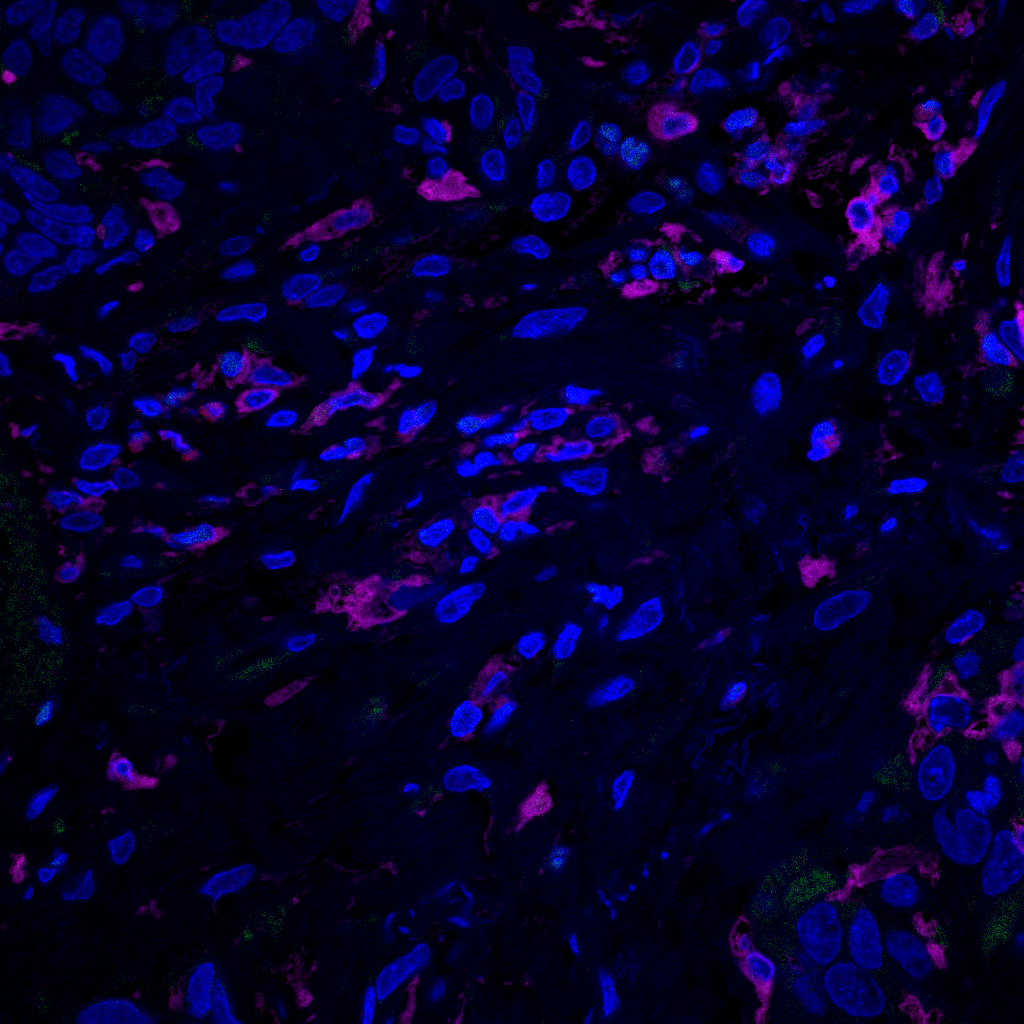

Supplement: Supplementary file 14 — Source data Fig. 3 [file 44321_2026_410_MOESM14_ESM.zip › Figure 3/3E/Representative Figure/20%/20%_c1-4.tif]

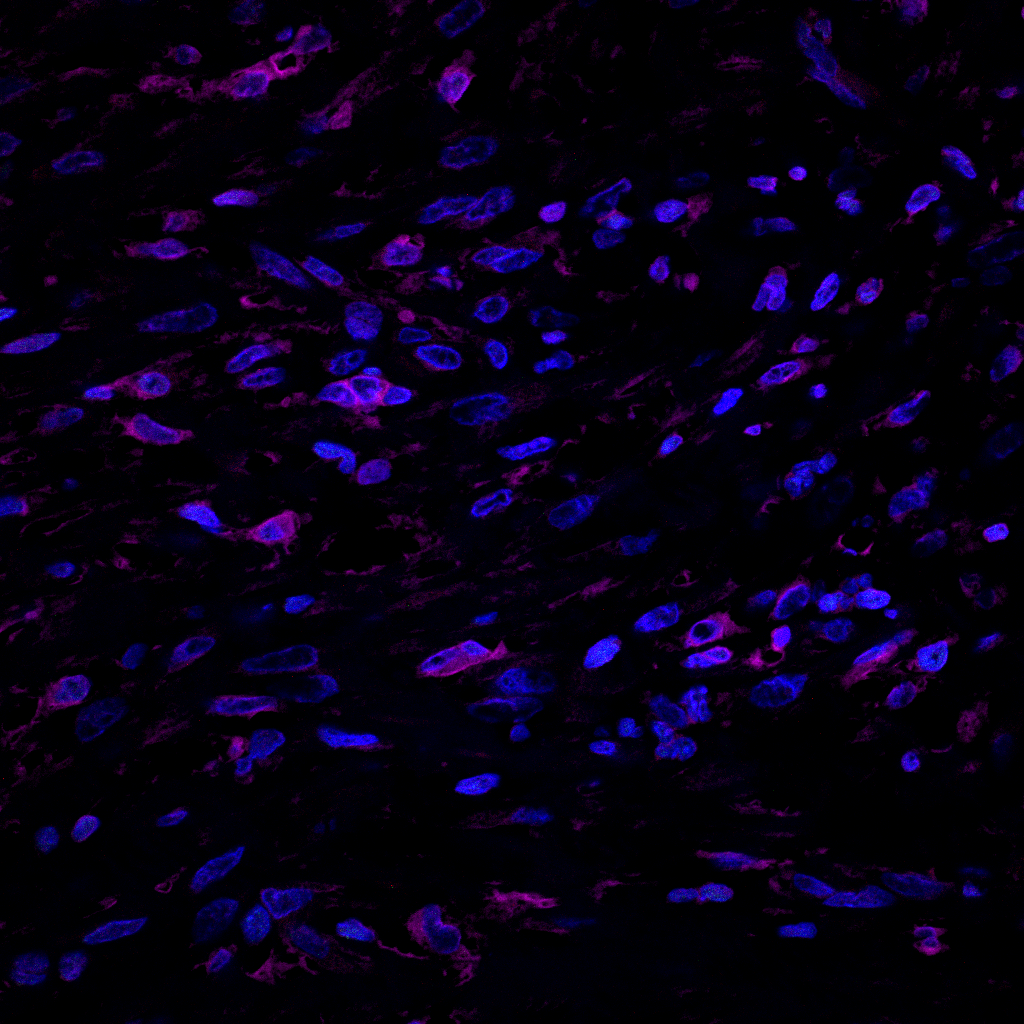

Supplement: Supplementary file 14 — Source data Fig. 3 [file 44321_2026_410_MOESM14_ESM.zip › Figure 3/3E/Representative Figure/60%/60%_c1-4.tif]
