# Supplementary material for: A monocyte-centered framework for predicting immunochemotherapy efficacy in lung squamous cell carcinoma patients
Source: EMBO Mol Med. 2026 Mar 30;18(5):1812–35. doi: 10.1038/s44321-026-00410-y (PMC13179367; doi:10.1038/s44321-026-00410-y)
Supplement: Supplementary file 15 — Expanded View Figures [file 44321_2026_410_MOESM15_ESM.pdf]

Expanded View Figures

Figure EV1. Cell-type annotation validation.

(**A**) Box plot and comparisons of smoking index between single-cell sequenced pCR/MPR and NMPR patients. Box plots followed the Tukey style (patient $n = 15$, pCR/MPR $n = 8$, NMPR $n = 7$; Centre line: median. Box bounds: 25th and 75th percentiles. Whiskers: extending to the most extreme data points within 1.5 times the interquartile range (IQR) from the box bounds. Outliers: points beyond the end of the whiskers). (**B**) UMAP of scRNA-seq data before removing batch effect. (**C**) Dot plot displaying marker gene expression used for major cell-type annotation. (**D**) Copy number variation scores compared between suspected tumor cells and other cells in the peripheral blood (blood sample $n_{blood} = 23$, before treatment $n_{pCR/MPR} = 5$, $n_{NMPR} = 4$, after treatment $n_{pCR/MPR} = 7$, $n_{NMPR} = 7$) and primary tumors (tumor sample $n_{tumor} = 26$, before treatment $n_{pCR/MPR} = 7$, $n_{NMPR} = 5$, after treatment $n_{pCR/MPR} = 7$, $n_{NMPR} = 7$). Comparison was conducted using Wilcoxon test (significant $p < 0.05$). Box plots followed the Tukey style (Centre line: median. Box bounds: 25th and 75th percentiles. Whiskers: extending to the most extreme data points within 1.5 times the interquartile range (IQR) from the box bounds. Outliers: points beyond the end of the whiskers). (**E**) IF staining of KRT5 (green) and DAPI (blue) of native tumor slices from patients in scRNA-seq cohort. (**F**) Box plot of major cell type proportion across different response groups (blood sample $n_{blood} = 23$, before treatment $n_{pCR/MPR} = 5$, $n_{NMPR} = 4$, after treatment $n_{pCR/MPR} = 7$, $n_{NMPR} = 7$; tumor sample $n_{tumor} = 26$, before treatment $n_{pCR/MPR} = 7$, $n_{NMPR} = 5$, after treatment $n_{pCR/MPR} = 7$, $n_{NMPR} = 7$). Box plots followed the Tukey style (Centre line: median. Box bounds: 25th and 75th percentiles. Whiskers: extending to the most extreme data points within 1.5 times the interquartile range (IQR) from the box bounds. Outliers: points beyond the end of the whiskers). (**G**) Comparison of major cell-type proportions excluding tumor cells (sample $n = 49$) with published LUSC scRNA-seq data (sample $n = 113$) before treatment. Comparison was conducted using Wilcoxon test (significant $p < 0.05$). Box plots followed the Tukey style (Centre line: median. Box bounds: 25th and 75th percentiles. Whiskers: extending to the most extreme data points within 1.5 times the interquartile range (IQR) from the box bounds. Outliers: points beyond the end of the whiskers).

 © The Author(s)

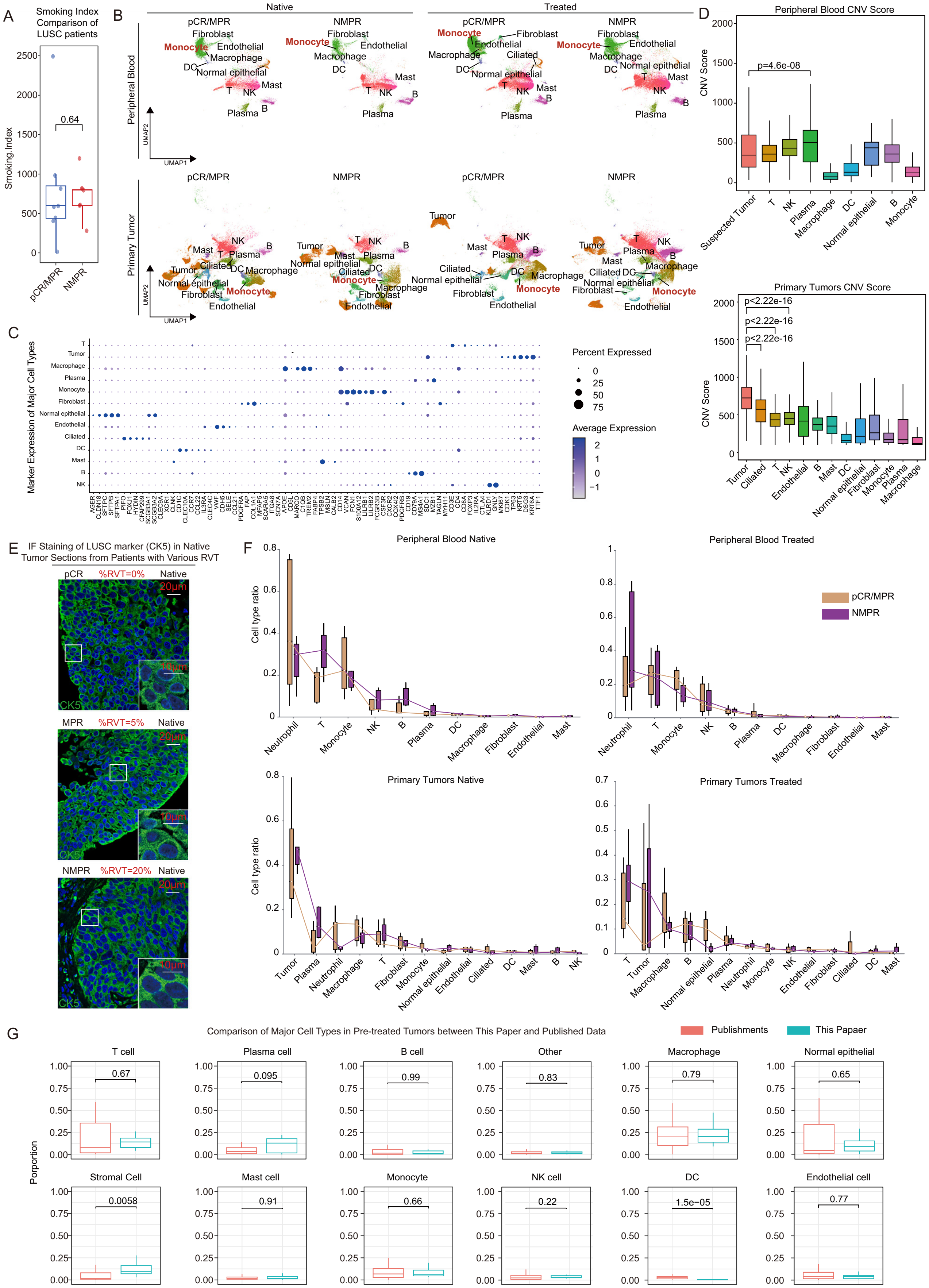
A
Smoking Index Comparison of LUSC patients
Smoking.Index
0.64
pCR/MPR
NMPR
B
Native
Treated
pCR/MPR
NMPR
Peripheral Blood
Primary Tumor
UMAP1
UMAP2
C
Marker Expression of Major Cell Types
Percent Expressed
Average Expression
D
Peripheral Blood CNV Score
CNV Score
p=4.6e-08
Primary Tumors CNV Score
p<2.22e-16
E
IF Staining of LUSC marker (CK5) in Native Tumor Sections from Patients with Various RVT
pCR %RVT=0% Native
MPR %RVT=5% Native
NMPR %RVT=20% Native
F
Peripheral Blood Native
Peripheral Blood Treated
Primary Tumors Native
Primary Tumors Treated
Cell type ratio
pCR/MPR
NMPR
G
Comparison of Major Cell Types in Pre-treated Tumors between This Paper and Published Data
Publishments
This Papaer
Proportion
T cell 0.67
Plasma cell 0.095
B cell 0.99
Other 0.83
Macrophage 0.79
Normal epithelial 0.65
Stromal Cell 0.0058
Mast cell 0.91
Monocyte 0.66
NK cell 0.22
DC 1.5e−05
Endothelial cell 0.77

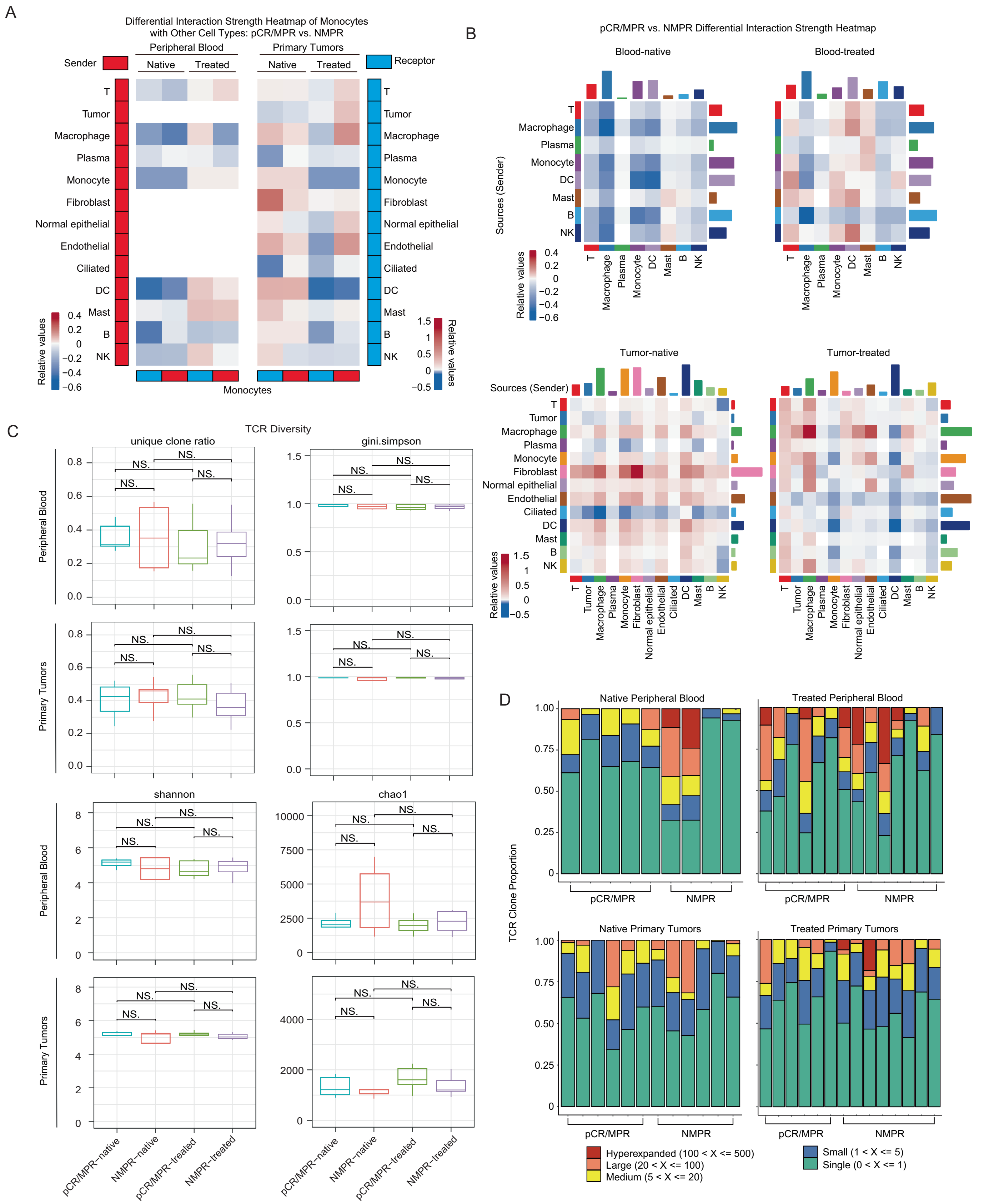
A
Differential Interaction Strength Heatmap of Monocytes with Other Cell Types: pCR/MPR vs. NMPR
Peripheral Blood
Primary Tumors
Native
Treated
Sender
Receptor
T
Tumor
Macrophage
Plasma
Monocyte
Fibroblast
Normal epithelial
Endothelial
Ciliated
DC
Mast
B
NK
Monocytes
Relative values
B
pCR/MPR vs. NMPR Differential Interaction Strength Heatmap
Blood-native
Blood-treated
Tumor-native
Tumor-treated
Sources (Sender)
C
TCR Diversity
unique clone ratio
gini.simpson
shannon
chao1
Peripheral Blood
Primary Tumors
NS.
pCR/MPR−native
NMPR−native
pCR/MPR−treated
NMPR−treated
D
Native Peripheral Blood
Treated Peripheral Blood
Native Primary Tumors
Treated Primary Tumors
TCR Clone Proportion
pCR/MPR
NMPR
Hyperexpanded (100 < X <= 500)
Large (20 < X <= 100)
Medium (5 < X <= 20)
Small (1 < X <= 5)
Single (0 < X <= 1)

 © The Author(s)

Figure EV2. Cell–cell communication heatmap and TCR diversity.

(**A**) pCR/MPR vs. NMPR differential interaction strength heatmap of major cell types. (**B**) Cell–cell communication difference heatmap among monocytes and other major cell types with pCR/MPR versus NMPR. (**C**) Comparison of four TCR diversity indicators among different response groups and between pre- and post-treatment groups (blood sample $n_{blood} = 23$, before treatment $n_{pCR/MPR} = 5$, $n_{NMPR} = 4$, after treatment $n_{pCR/MPR} = 7$, $n_{NMPR} = 7$; tumor sample $n_{tumor} = 26$, before treatment $n_{pCR/MPR} = 7$, $n_{NMPR} = 5$, after treatment $n_{pCR/MPR} = 7$, $n_{NMPR} = 7$). Comparison was conducted using Wilcoxon test (significant $p < 0.05$). Box plots followed the Tukey style (Centre line: median. Box bounds: 25th and 75th percentiles. Whiskers: extending to the most extreme data points within 1.5 times the interquartile range (IQR) from the box bounds. Outliers: points beyond the end of the whiskers). (**D**) Relative proportions of specific TCR clonotypes across samples; TCR clonotypes were determined based on clonotype expansion levels: Single (one occurrence); Small (>1 and ≤5); Medium (>5 and ≤20); Large (>20 and ≤100); Hyperexpanded (>100).

© The Author(s)

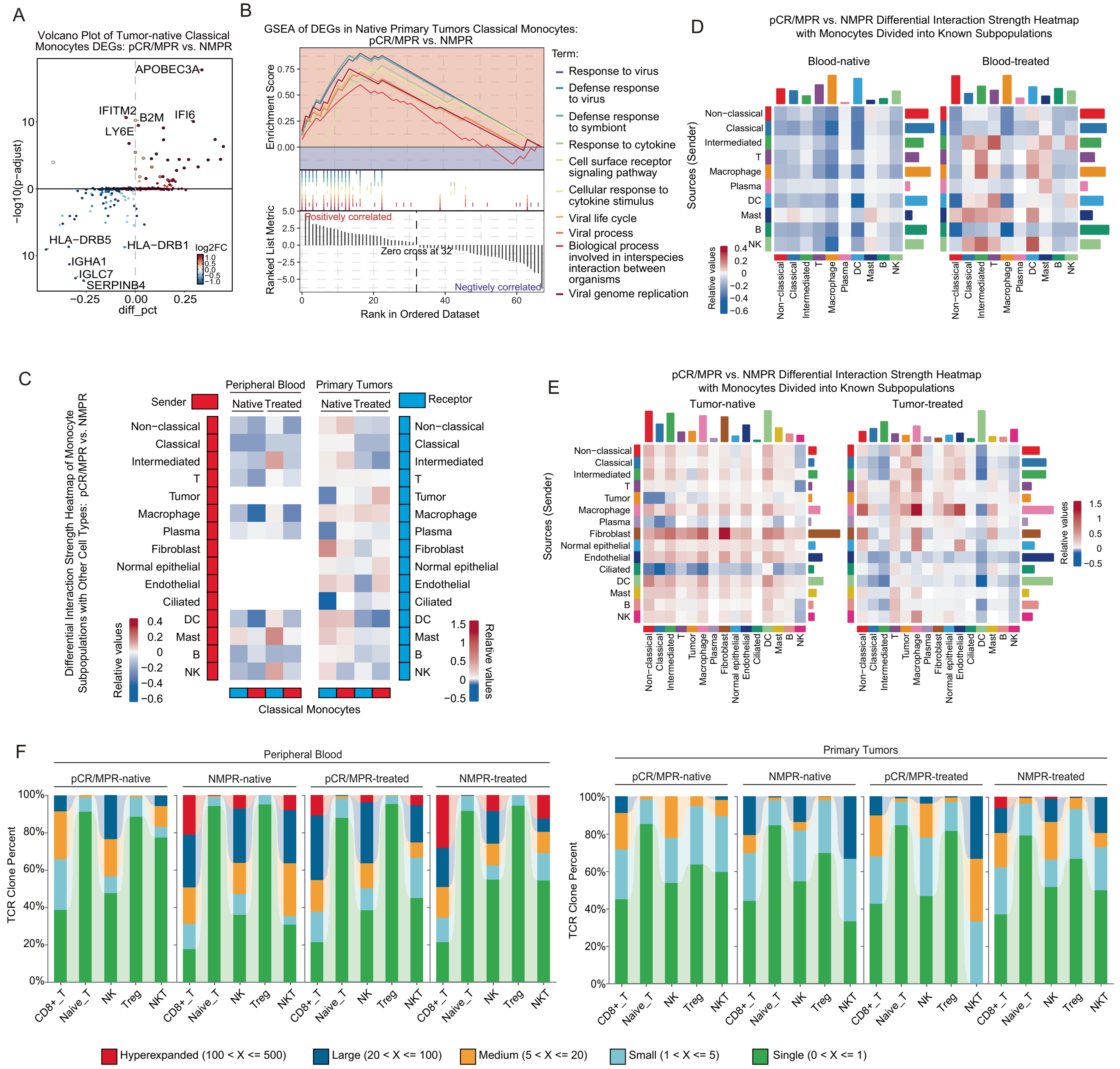

Figure EV3. GSEA and cell–cell communication of monocyte subpopulations before and after treatment.

(**A**) Volcano plot of DEGs with pCR/MPR versus NMPR classical monocytes in native tumors (native tumor sample $n_{tumor} = 12$, $n_{pCR/MPR} = 7$, $n_{NMPR} = 5$); the x-axis indicates percentage differences in gene-expressing cells between groups, and the y-axis shows $-\log_{10}$(adjusted *p*-value). Dots are colored based on $\log_2$FC values. Two-sided Wilcoxon test was utilized to calculate *p*-values and *p*-values were adjusted by Bonferroni correction. (**B**) GSEA plot of top 10 upregulated pathways based on DEGs in Fig. EV3A. (**C**) Cell–cell communication difference heatmap among classical monocytes and other major cell types with pCR/MPR versus NMPR. (**D**) Peripheral blood pCR/MPR vs. NMPR differential interaction strength heatmap of major cell types with monocytes divided into three subpopulations. (**E**) Primary tumor pCR/MPR vs. NMPR differential interaction strength heatmap of major cell types with monocytes divided into three subpopulations. (**F**) Relative proportions of specific TCR clonotypes among T cell subpopulations across patient groups; TCR clonotypes were determined based on clonotype expansion levels: Single (one occurrence); Small (>1 and ≤5); Medium (>5 and ≤20); Large (>20 and ≤100); Hyperexpanded (>100); T cell subpopulation abbreviations: CD8 + T_{EMRA}/T_{EFF}: effector memory or effector T cells; CD8 + T_{CM}: CD8+ central memory T cells; T_N: naive T cells; CD4+ Normal-T_{CM}: CD4$^+$ Normal-central memory T cells; CD4+ Blood-T_{CM}: CD4$^+$ Blood-central memory T cells; Tumor-T_{reg}: tumor-infiltrating T regulatory; CD8 + T_{EX}: exhausted CD8$^+$ T cells; CD4 + T_{RM}: tissue-resident memory T cells.

 © The Author(s)

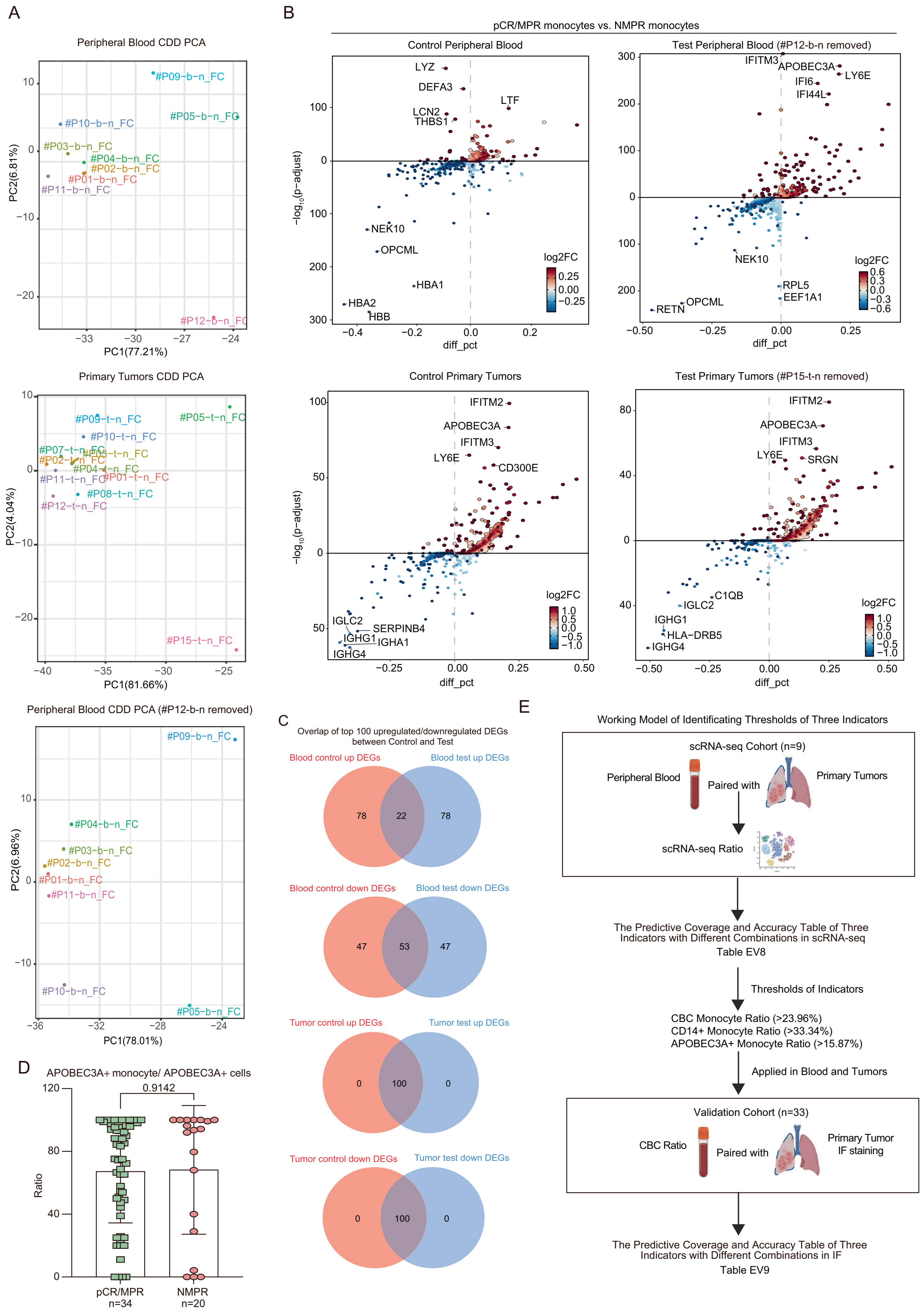
A
Peripheral Blood CDD PCA
PC1(77.21%)
PC2(6.81%)
Primary Tumors CDD PCA
PC1(81.66%)
PC2(4.04%)
Peripheral Blood CDD PCA (#P12-b-n removed)
PC1(78.01%)
PC2(6.96%)
B
pCR/MPR monocytes vs. NMPR monocytes
Control Peripheral Blood
Test Peripheral Blood (#P12-b-n removed)
Control Primary Tumors
Test Primary Tumors (#P15-t-n removed)
−log10(p−adjust)
diff_pct
log2FC
C
Overlap of top 100 upregulated/downregulated DEGs between Control and Test
Blood control up DEGs
Blood test up DEGs
78
22
78
Blood control down DEGs
Blood test down DEGs
47
53
47
Tumor control up DEGs
Tumor test up DEGs
0
100
0
Tumor control down DEGs
Tumor test down DEGs
0
100
0
D
APOBEC3A+ monocyte/ APOBEC3A+ cells
0.9142
Ratio
pCR/MPR n=34
NMPR n=20
E
Working Model of Identificating Thresholds of Three Indicators
scRNA-seq Cohort (n=9)
Peripheral Blood
Paired with
Primary Tumors
scRNA-seq Ratio
The Predictive Coverage and Accuracy Table of Three Indicators with Different Combinations in scRNA-seq
Table EV8
Thresholds of Indicators
CBC Monocyte Ratio (>23.96%)
CD14+ Monocyte Ratio (>33.34%)
APOBEC3A+ Monocyte Ratio (>15.87%)
Applied in Blood and Tumors
Validation Cohort (n=33)
CBC Ratio
Paired with
Primary Tumor IF staining
The Predictive Coverage and Accuracy Table of Three Indicators with Different Combinations in IF
Table EV9

Figure EV4. Imitating case deletion diagnosis analysis of native peripheral blood and primary tumors.

(**A**) PCA dimensionality reduction of imitating case deletion diagnosis (CDD) in all native blood, all native tumor and native blood samples with #P12-b-n sample removed. Each point indicates the DEG fold change profiling of native pCR/MPR versus NMPR monocytes after one sample removed. The points are named by the removed samples. Fox example, P12-b-n_FC means that we removed #P12-b-n sample from pCR/MPR native blood and calculated DEGs fold changes with rest pCR/MPR versus NMPR native blood monocytes. (**B**) Volcano plot of DEGs with native pCR/MPR versus NMPR monocytes. Control means all native pCR/MPR versus NMPR samples (native blood sample $n_{blood} = 9$, $n_{pCR/MPR} = 5$, $n_{NMPR} = 4$; native tumor sample $n_{tumor} = 12$, before treatment $n_{pCR/MPR} = 7$, $n_{NMPR} = 5$). Test means all pCR/MPR versus NMPR with outlier samples removed (removed sample ID in title; native blood sample $n_{blood} = 8$, $n_{pCR/MPR} = 5$, $n_{NMPR} = 3$; native tumor sample $n_{tumor} = 11$, before treatment $n_{pCR/MPR} = 7$, $n_{NMPR} = 4$). Two-sided Wilcoxon test was utilized to calculate *p*-values and *p*-values were adjusted by Bonferroni correction. (**C**) Top 100 upregulated and top 100 downregulated DEGs overlap between Control and Test results in (**B**). (**D**) The ratio of APOBEC3A+ monocyte versus APOBEC3A+ cells based on IF staining (pCR/MPR $n = 34$, NMPR $n = 20$; two-sided T-test, significant $p < 0.05$). Error bars of bar plots represented the standard deviation. (**E**) Working model of identificating thresholds of three indicators.

 © The Author(s)

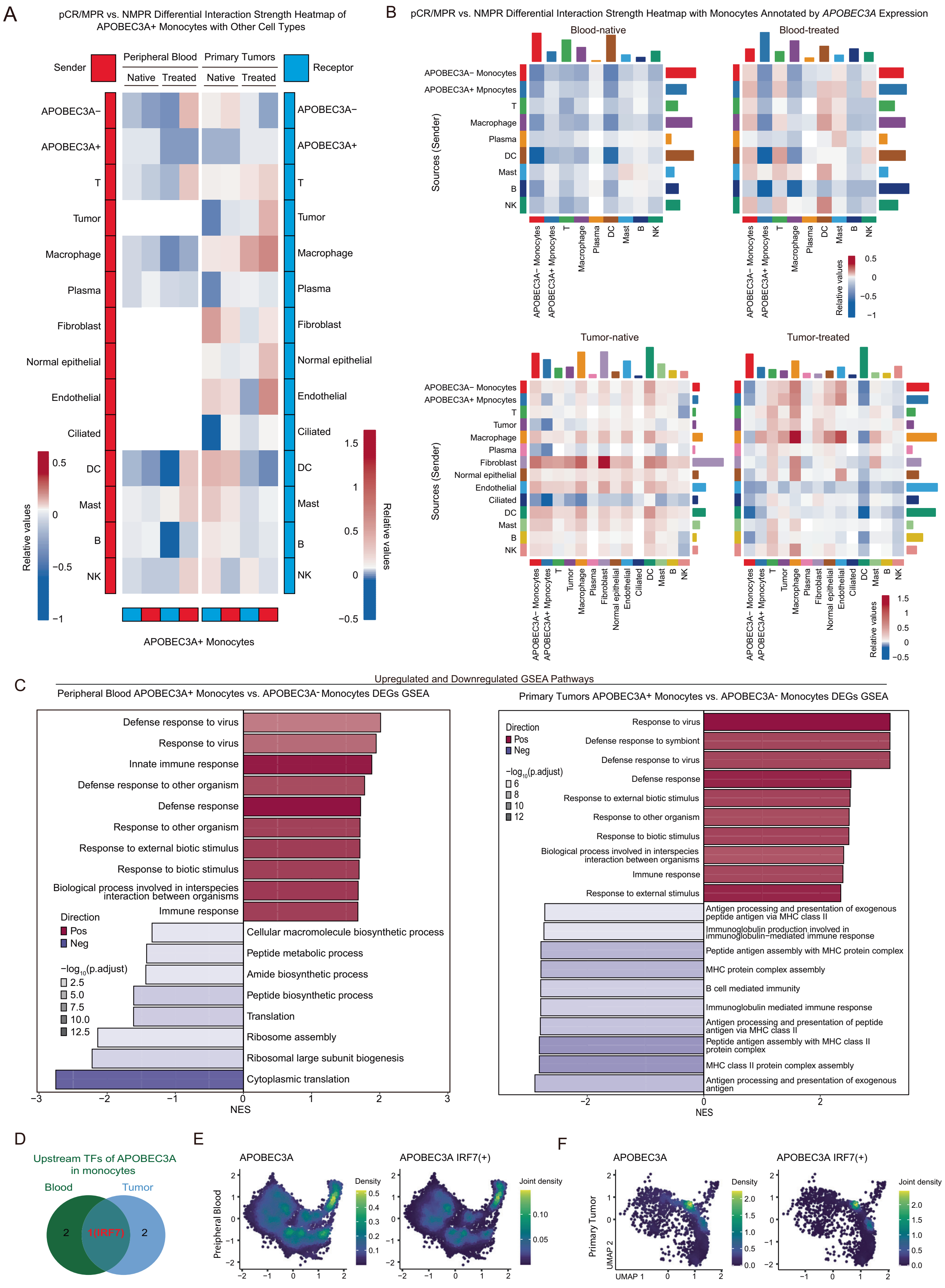
A
pCR/MPR vs. NMPR Differential Interaction Strength Heatmap of APOBEC3A+ Monocytes with Other Cell Types
Sender
Peripheral Blood
Primary Tumors
Native
Treated
Native
Treated
Receptor
APOBEC3A−
APOBEC3A+
T
Tumor
Macrophage
Plasma
Fibroblast
Normal epithelial
Endothelial
Ciliated
DC
Mast
B
NK
Relative values
APOBEC3A+ Monocytes
B
pCR/MPR vs. NMPR Differential Interaction Strength Heatmap with Monocytes Annotated by APOBEC3A Expression
Blood-native
Blood-treated
Tumor-native
Tumor-treated
Sources (Sender)
APOBEC3A− Monocytes
APOBEC3A+ Mpnocytes
Relative values
C
Upregulated and Downregulated GSEA Pathways
Peripheral Blood APOBEC3A+ Monocytes vs. APOBEC3A- Monocytes DEGs GSEA
Defense response to virus
Response to virus
Innate immune response
Defense response to other organism
Defense response
Response to other organism
Response to external biotic stimulus
Response to biotic stimulus
Biological process involved in interspecies interaction between organisms
Immune response
Cellular macromolecule biosynthetic process
Peptide metabolic process
Amide biosynthetic process
Peptide biosynthetic process
Translation
Ribosome assembly
Ribosomal large subunit biogenesis
Cytoplasmic translation
Direction
Pos
Neg
−log10(p.adjust)
NES
Primary Tumors APOBEC3A+ Monocytes vs. APOBEC3A- Monocytes DEGs GSEA
Response to virus
Defense response to symbiont
Defense response to virus
Defense response
Response to external biotic stimulus
Response to other organism
Response to biotic stimulus
Biological process involved in interspecies interaction between organisms
Immune response
Response to external stimulus
Antigen processing and presentation of exogenous peptide antigen via MHC class II
Immunoglobulin production involved in immunoglobulin-mediated immune response
Peptide antigen assembly with MHC protein complex
MHC protein complex assembly
B cell mediated immunity
Immunoglobulin mediated immune response
Antigen processing and presentation of peptide antigen via MHC class II
Peptide antigen assembly with MHC class II protein complex
MHC class II protein complex assembly
Antigen processing and presentation of exogenous antigen
D
Upstream TFs of APOBEC3A in monocytes
Blood
Tumor
2
1(IRF7)
2
E
APOBEC3A
APOBEC3A IRF7(+)
Preipheral Blood
Density
Joint density
F
APOBEC3A
APOBEC3A IRF7(+)
Primary Tumor
UMAP 2
UMAP 1
Density
Joint density

Figure EV5. GSEA analysis, cell–cell interaction and upstream analysis of APOBEC3A+/- monocytes of LUSC patients.

(**A**) Cell–cell communication difference heatmap among APOBEC3A+/- monocytes and other major cell types with pCR/MPR versus NMPR. (**B**) pCR/MPR vs. NMPR differential interaction strength heatmap with monocytes annotated by APOBEC3A expression. (**C**) GSEA pathway bar plot showing upregulated and downregulated DEGs of APOBEC3A+ versus APOBEC3A- monocytes in blood and tumors. (**D**) Overlap of upstream TFs of APOBEC3A in native blood and tumor monocytes. (**E**) Expression density of APOBEC3A; Co-expression density of APOBEC3A and IRF7(+) in native blood monocytes. (**F**) Expression density of APOBEC3A; Co-expression density of APOBEC3A and IRF7(+) in native tumor monocytes.

 © The Author(s)
